# Supplementary material for: Network-based Biased Tree Ensembles (NetBiTE) for Drug Sensitivity Prediction and Drug Sensitivity Biomarker Identification in Cancer
Source: Sci Rep. 2019 Nov 4;9:15918. doi: 10.1038/s41598-019-52093-w (PMC6828742; doi:10.1038/s41598-019-52093-w)
Supplement: Supplementary file 1 — Supplementary Document [file 41598_2019_52093_MOESM1_ESM.pdf]

Network-based Biased Tree Ensembles (NetBiTE) for Drug Sensitivity Prediction and Drug Sensitivity Biomarker Identification in Cancer

Ali Oskooei[1], Matteo Manica[1,2], Roland Mathis[1] and Maria Rodriguez Martinez[1]

[1] IBM Research – Zurich, Säumerstrasse 4, 8803 Rüschlikon, Switzerland

[2] Institute für Molekulare Systembiologie, Auguste-Piccard-Hof 1 8093, Zürich, Switzerland

S1 Characterization of tuning parameters

In this study, we swept across a range of values for number of trees (n_{tree}), *mtry* and the target partition size (*TPS*) to investigate how sensitive our model is to variations in each of these parameters when applying regression tree ensemble algorithms to the Genomics of Drug Sensitivity in Cancer (GDSC) (1) dataset. The tuning parameter characterization study was performed for a panel of 50 cancer drugs tested against 883 cell lines.

We studied the model performance with the number of trees ranging from 10 to 5000 as shown in figure S1B. As presented in the plot, beyond n_{tree} = 50 the model performance is approximately flat with a p-value of 0.75 given by a one-way ANOVA test. Beyond 500 trees there is no detectable variation in model performances and as a result we adopted n_{tree} = 500 as the optimal number of trees in our tree ensemble models.

In figure S1C, we studied the effect of *mtry* on model performance. We varied *mtry* from 10 to 5233, one-third of the total number of features (genes), which is the recommended mtry for RF regression in various sources in the literature (2,3). As shown in the plot the model performance does not vary across different *mtry* values with a p-value of 0.4 given by a one-way ANOVA test. This observation suggests that given the GDSC dataset, the choice of *mtry* within the recommended range, does not improve the

model accuracy significantly. On the other hand, the choice of a high *mtry* is computationally expensive, as thousands of features need to be considered by the model rendering the model computationally inefficient. As such, the lowest possible *mtry* that results in a maximal model accuracy must be selected.

The effect of various target partition sizes (*TPS*) ranging from 1 to 100 was studied. As shown in the plot the RF algorithm is not sensitive to *TPS* as indicated by a p-value of 0.98 given by a one-way ANOVA test. As a result, we adopted *TPS* = 1 throughout this work as it resulted in the highest mean accuracy compared to other *TPS* values studied, however only by a slight margin.

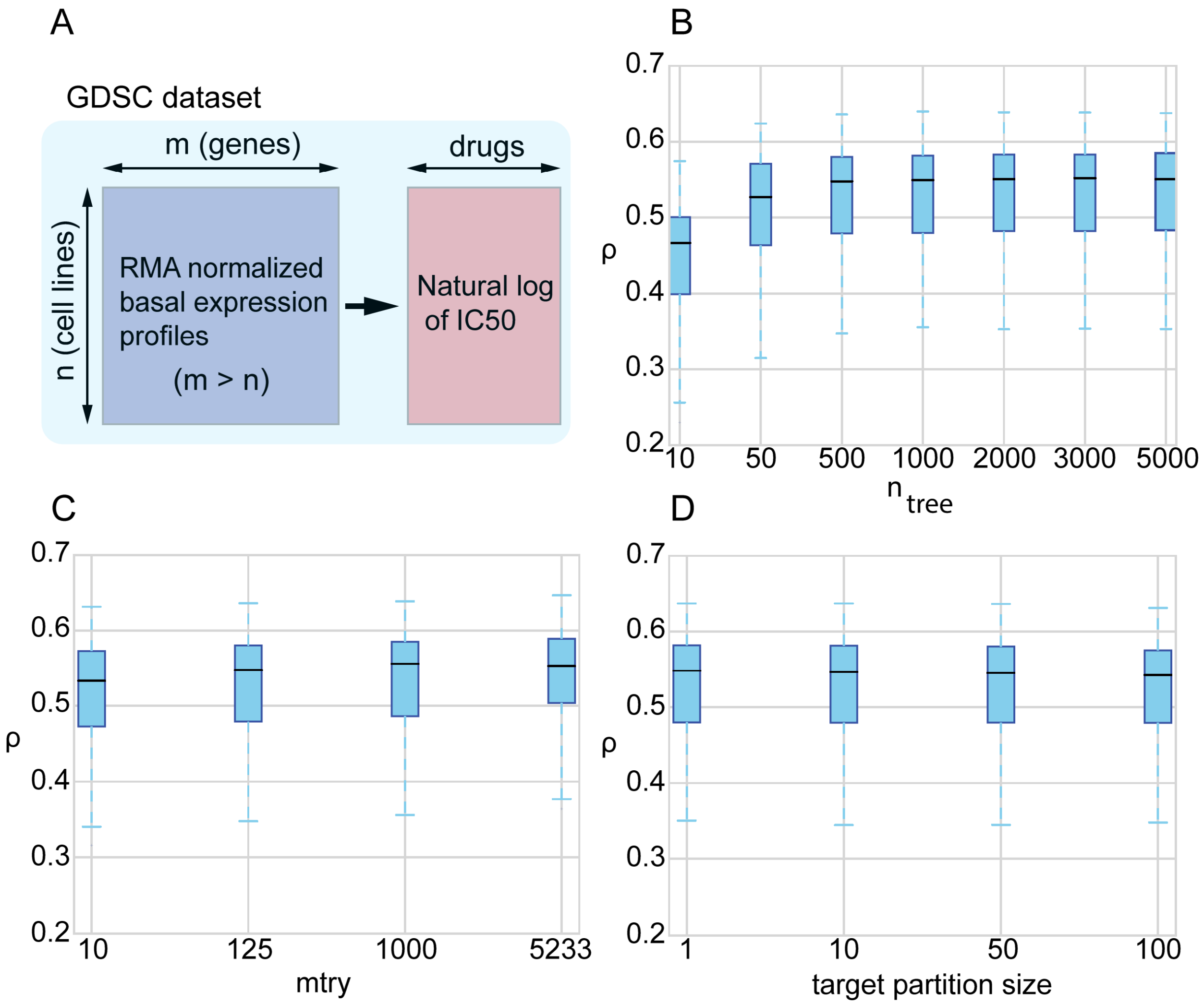

Figure S1. A) to train and test our models we used the drug sensitivity screening data available at Genomics of Drug Sensitivity in Cancer (GDSC) database (1). In our model, we incorporated RMA-normalized basal expression

profiles of *n* = 883 cancer cell lines tested with 50 compounds as well as the natural logarithm of IC50 for each compound tested with each cell line. B) using the GDSC dataset we trained a RF model with various number of trees (n_{tree}) while keeping *mtry* fixed at *mtry* = 125. As shown in the plot beyond 50 trees the model performance (Pearson correlation coefficients, ρ) plateaus with a p-value of 0.75 given by a one-way ANOVA test. C) Fixing the number of trees at n_{tree} = 500, we investigated the effect of varying the number of features considered at each split, *mtry*, from *mtry* = 10 features to *mtry* = 5233 features. As shown in the plot the model performance was not sensitive to the choice of *mtry* as indicated by a one-way ANOVA test returning a p-value of 0.4. D) the effect of target partition size or the number of samples in each leaf node (n_{tree} = 500 and *mtry* = 125), was investigated and was not found to impact the model significantly with a p-value of 0.98 given by a one-way ANOVA test. As a result, *TPS* = 1 was adopted throughout this work.

S2 Investigation of propagation rate

In figure S2, we investigated the effect of various α (i.e., the tuning parameter for the network diffusion depth of prior knowledge weights over the STRING network) for the drug Cabozantinib. As shown in the figure with a gradual increase in α from α = 0, the model accuracy increases until it peaks at α = 0.7. Any further increase in α past the optimal value of 0.7 results in a decrease in model accuracy. With this experiment, we confirmed that the optimal value of α = 0.7, as reported in literature for the STRING network, is valid. In addition, in this experiment we compared NetBiTE accuracy with the accuracy of RF and linear regression (LR) and observed that NetBiTE outperforms both RF and LR with a significant margin as shown in the figure S2.

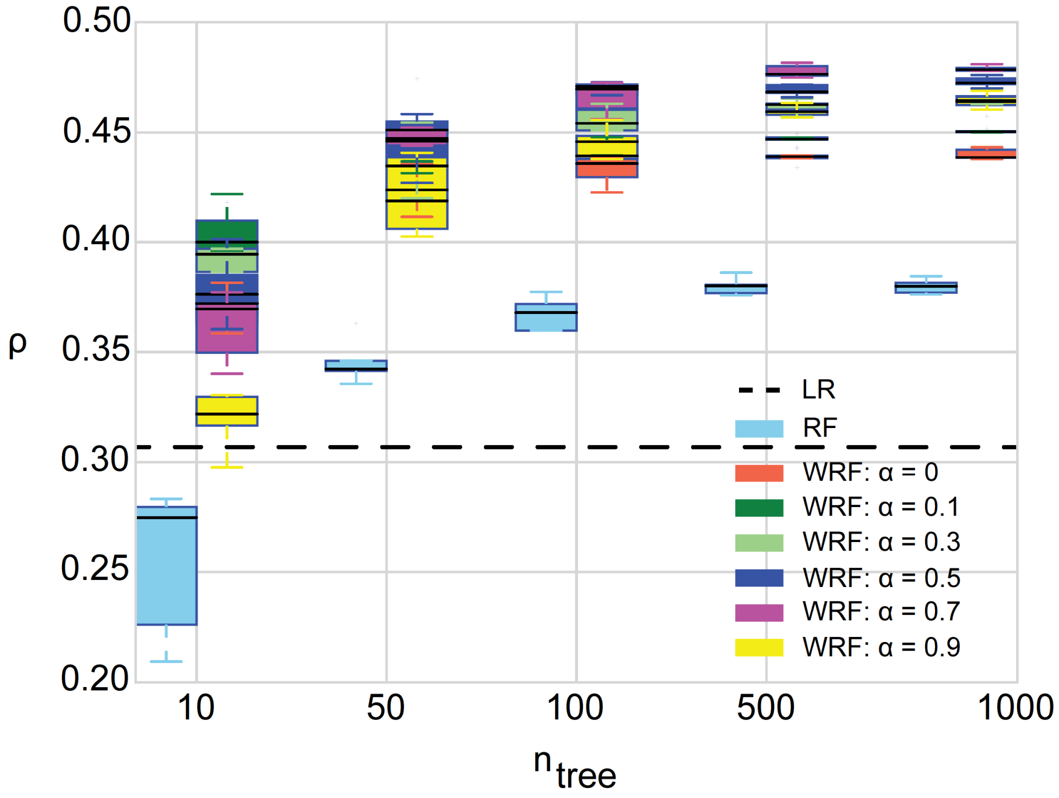

Figure S2. The study of variation in the network propagation tuning parameter *α* for Cabozantinib, one of the drugs that showed improvement with NetBiTE. As shown in the figure, the value for *α* was varied from 0 to 0.9 and the model performance was compared to standard RF and linear regression (LR). The model performance continuously increased with an increase in *α* and peaked at *α* = 0.7. For *α* > 0.7 NetBiTE performance dropped and at *α* = 1 the weight vector was essentially uniform with equal weights for all genes resulting in a NetBiTE performance that is identical to standard RF. As such, we confirmed *α* = 0.7 as the optimal value and chose this value throughout our studies.

S3 Analysis of drug target genes

In order to determine which membrane receptor genes are more likely to be responsible for the accuracy improvements observed using NetBiTE (see figure 4), we plotted the top three genes and their frequency of occurrence in responsive and non-responsive drugs within each category. For RTK signaling pathway inhibitors (RSPIs), there is a clear discrepancy between the most frequent genes in responsive versus non-responsive drugs, as shown in figure S3A and S3B. KIT, FLT3 and PDGFRB are the most frequent targets in responsive drugs while KDR, TGFBR1 and FGFR1 are most frequent targets in non-responsive RSPI drugs. For EGFR signaling pathway inhibitors (ESPIs), ERBB2 is a target exclusively in

responsive drugs. For IGFR signaling pathway inhibitors, INSRR is a frequent target, exclusively in responsive drugs.

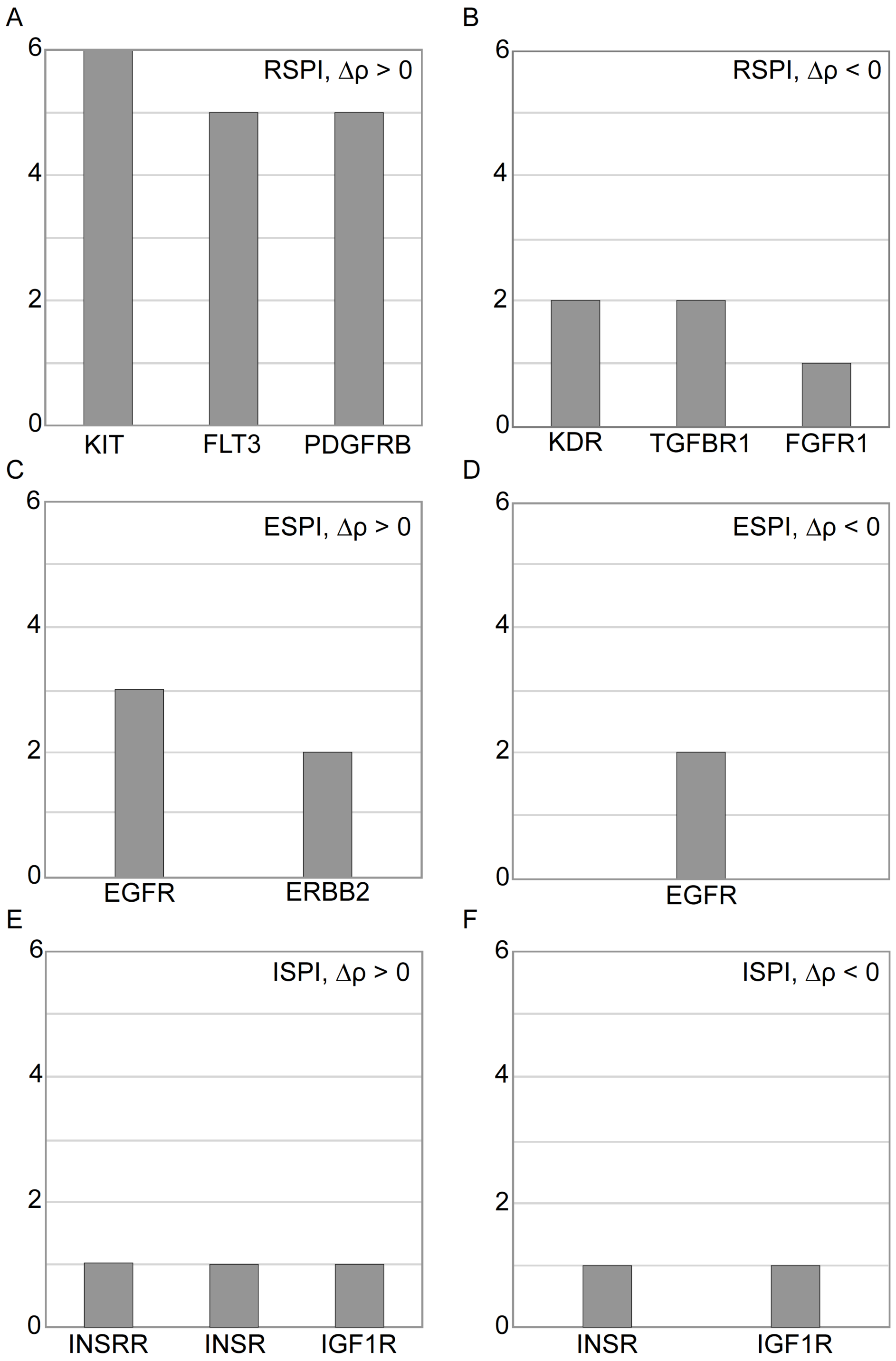

Figure S3. Top-three most frequent target genes in drugs that are responsive ($\Delta\rho > 0$) and non-responsive ($\Delta\rho < 0$) to NetBiTE for the three categories of membrane receptor inhibitor drugs studied. The values on the y-axis are the

number of drugs that had the given gene as a target. A) Most frequent genes among responsive RTK signaling pathway inhibitors. B) Most frequent genes among non-responsive RTK signaling pathway inhibitors. C) Most frequent genes among responsive EGFR signaling pathway inhibitors. D) Most frequent genes among non-responsive EGFR signaling pathway inhibitors. E) Most frequent genes among responsive IGFR signaling pathway inhibitors. F) Most frequent genes among non-responsive IGFR signaling pathway inhibitors.

References

1. Yang W, Soares J, Greninger P, Edelman E, Lightfoot H, Forbes S, et al. Genomics of Drug Sensitivity in Cancer (GDSC): a resource for therapeutic biomarker discovery in cancer cells. Nucleic Acids Res. 2013; 41(Database issue):D955–61.

2. Hastie T, Tibshirani R, Friedman J. The Elements of Statistical Learning: Data Mining, Inference, and Prediction, Second Edition [Internet]. Springer New York; 2009. (Springer Series in Statistics).

3. Breiman L. Random Forests. Mach Learn [Internet]. 2001; 45. Available from: http://dx.doi.org/10.1023/A:1010933404324
